# Farnesoid X Receptor as Target for Therapies to Treat Cholestasis-Induced Liver Injury

**DOI:** 10.3390/cells10081846

**Published:** 2021-07-21

**Authors:** Anca D. Petrescu, Sharon DeMorrow

**Affiliations:** 1 Division of Pharmacology and Toxicology, College of Pharmacy, The University of Texas at Austin, Austin, TX 78712, USA; anca.petrescu@austin.utexas.edu; 2 Department of Internal Medicine, Dell Medical School, The University of Texas at Austin, Austin, TX 78712, USA; 3 Central Texas Veterans Health Care System, Temple, TX 78712, USA

**Keywords:** FXR, liver, cholestasis, obeticholic acid, rifampicin, autophagy

## Abstract

Recent studies on liver disease burden worldwide estimated that cirrhosis is the 11th most common cause of death globally, and there is a great need for new therapies to limit the progression of liver injuries in the early stages. Cholestasis is caused by accumulation of hydrophobic bile acids (BA) in the liver due to dysfunctional BA efflux or bile flow into the gall bladder. Therefore, strategies to increase detoxification of hydrophobic BA and downregulate genes involved in BA production are largely investigated. Farnesoid X receptor (FXR) has a central role in BA homeostasis and recent publications revealed that changes in autophagy due to BA-induced reactive oxygen species and increased anti-oxidant response via nuclear factor E2-related factor 2 (NRF2), result in dysregulation of FXR signaling. Several mechanistic studies have identified new dysfunctions of the cholestatic liver at cellular and molecular level, opening new venues for developing more performant therapies.

## 1. Introduction

According to studies on the burden of liver diseases worldwide, it is estimated that approximately 1 million people die each year due to complications of cirrhosis, and 1 million due to viral hepatitis and hepatocellular carcinoma [1]. Cirrhosis is currently the 11th most common cause of death globally, and liver cancer is the 16th leading cause of death. For patients with end-stage liver disease, the only available therapy is liver transplantation, and at the present rate, less than 10% of transplantation needs are met [1]. A common denominator of a large spectrum of hepatic diseases is liver fibrosis, and therefore a robust number of research studies aim to develop new therapies to mitigate this condition.

Cholestasis is a clinical syndrome with intra- or extra-hepatic etiology, resulting from the obstruction of bile secretion and flow from the liver into the gall bladder and duodenum [2]. Cholestasis can be caused by mutations of genes encoding for proteins with roles in bile transport from hepatocytes into cholangiocytes and bile ducts, resulting in the retention of BA in the liver. The bile flow can also be obstructed by gall stones due to metabolic dysfunctions (cholelithiasis), by tumors (hepatocellular carcinoma, cholangiocarcinoma), or by parasitic infections [2]. Other causes of cholestasis include immune-mediated conditions such as primary sclerosing cholangitis (PSC), primary biliary cirrhosis (PBC), and also exposure to certain medications which negatively affect the liver (non-steroidal anti-inflammatory drugs, anti-diabetic medications). Numerous cholestatic disorders are chronic and may lead to liver fibrosis and cirrhosis if left untreated.

Cholestatic liver injury is a complex disease with a multitude of dysfunctions including not only BA metabolism and transport, but also excessive cell proliferation especially of cholangiocytes and hepatic stellate cells (HSC) which become activated and initiate signaling pathways involving anti-oxidant and immune responses. Therefore, the mechanistic studies on cholestasis encompass a very large area starting with dysregulation of BA metabolism, transport, and signaling on parenchymal and non-parenchymal cells and expanding to inflammation, fibrosis, and eventually carcinogenesis.

BA have critical roles in the regulation of a multitude of physiological processes related to nutrition and digestion, mediating the transport and metabolism of lipids, influencing glucose and insulin sensitivity and modulating the overall energy expenditure in the body [3,4]. BA are signaling molecules acting on receptors that have been defined as BA sensors, and are from two different classes of receptors: (i) nuclear receptors such as farnesoid X receptor (FXR; also known as NR1H4), pregnane X receptor (PXR), constitutive androstane receptor (CAR), and vitamin D receptor (VDR), that become activated by BA and bind to specific response elements on target genes influencing the rate of their transcription; and (ii) membrane-bound G-protein coupled receptors such as Takeda G-protein receptor 5 (TGR5) or G protein-coupled BA receptor 1 (GPBAR1) and spingosine-1-phosphate receptor 2 (S1PR2). In this review we focus on FXR since it is the main player in dysregulation of BA homeostasis in the context of cholestatic injury of the liver. Known therapies as well as new potential drug targets from mechanistic studies on cholestasis-induced liver fibrosis, with focus on dysfunctions in metabolism and transport of hydrophobic BA, are outlined. Several molecular and cellular signaling pathways initiated by FXR in the liver are described. Special attention is also given to a possible role of FXR in autophagy in the context of hepatic cholestasis, bringing light on new potential drug targets for more efficient therapies.

## 2. FXR

Numerous studies on FXR^−/−^ mice revealed essential roles of FXR in liver functions including the regulation of BA, lipid, and glucose homeostasis [5]. FXR activators have been successfully applied for treatment of dyslipidemia, insulin resistance, and steatosis in obesity, diabetes, alcohol-induced liver injury, and non-alcohol fatty liver or steatohepatitis [5,6,7]. However, the role of FXR in cholestasis in animal models or clinical studies is still unclear and controversial. Cholestatic liver disorders have a large range of different etiologies resulting in reduced bile flow and interruption of enterohepatic circulation, leading to accumulation of toxic bile, rich in hydrophobic BA in the liver. Excessive BA in the liver cause hepatocyte damage, activation of Kupffer cells, HSC, and cholangiocytes, followed by hepatic inflammation and liver fibrosis.

**Figure 1:**
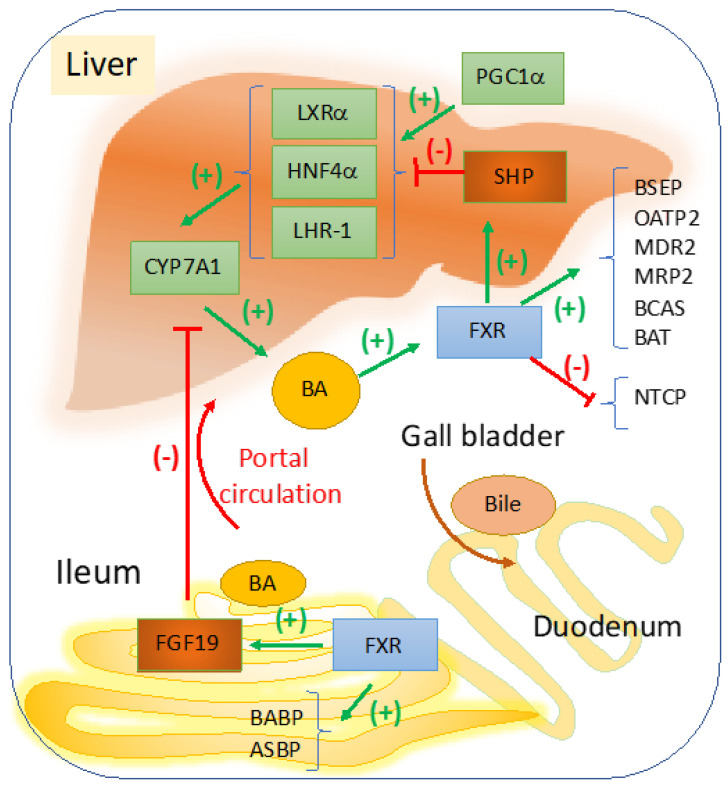
FXR regulation of bile acid synthesis, transport and hepato-enteric circulation. Cyp7a1 is the rate limiting enzyme in BA synthesis, and it is transactivated by LXRα, HNF4α, and LHR-1 which in turn are stimulated by various ligands derived from lipid nutrients including cholesterol (for LXRα), fatty acyl-CoA (for HNF4α), dietary phospholipids for LRH-1 or liver receptor homologue-1 [18,19]. When BA becomes abundant in the liver, a negative feedback loop starts to function via FXR to maintain balance and to control the elimination of excess BA. BA-induced activation of FXR results in upregulation of SHP which is a co-repressor of LXRα, HNF4α, and LHR-1. FXR upregulates genes with role in BA conjugation, i.e., BA CoA synthase (BCAS) and BA CoA: amino acid N-acetyltransferase (BAT). FXR mediates the bile formation and flow from the liver to the gallbladder by upregulating bile salt export pump (BSEP, ABCB11) which exports BA from hepatocytes; multidrug resistance associated 2 (MRP2, ABCC2) for the transport of BA amongst many other components of the bile into the gallbladder. Other proteins such as multidrug resistance protein 2 (MDR2), a transporter of phospholipids, and ABCG5, ABCG8 that transport cholesterol to the gallbladder are also upregulated by FXR. In the intestine, FXR transactivates ileum BA binding protein (IBABP) and mediates BA reabsorption by apical sodium-dependent bile salt transporter (ASBT). From portal circulation BA are reabsorbed into hepatocytes via sodium-dependent taurocholate cotransport peptide (NTCP), and organic anion transport protein 2 (OATP2). In the intestine, BA-activated FXR upregulates FGF19, a growth factor that is secreted into the portal circulation and inhibits Cyp7a1-controled BA synthesis in hepatocytes. BA synthesis is upregulated when BA concentration in the liver is low and SHP is replaced by PGC1α co-activator of genes that stimulate Cyp7a1 synthesis [11].

FXR is the main sensor and regulator of BA metabolism in the liver and intestine, with a role in maintaining BA homeostasis during the daily cycles of feeding [8,9]. In Figure 1, a schematic representation describes the most important signaling pathways with impact in cholestasis, that are induced by changes in BA concentrations within the liver versus ileum. A more comprehensive illustration of the overall effects of FXR in liver diseases, including modulation of inflammation, portal hypertension, and carcinogenesis, was published before by Fuchs et al. [10]. In the liver, FXR represses genes involved in BA synthesis via small heterodimer partner (SHP) which is a co-repressor that competes with peroxisome proliferator activated receptor gamma coactivator 1 alpha (PGC1α) which activates genes that stimulate BA synthesis [11]. Overall, FXR in the liver and ileum control BA homeostasis modulating genes of BA synthesis and transport. Therefore, several cholestatic diseases are associated with dysfunctions in signaling pathways regulated by FXR.

Genetic studies revealed several defects of genes involved in FXR signaling pathway in cases of pediatric and adult cholestasis [12]. Thus, in intrahepatic cholestasis of pregnancy (ICP), a disease that occurs in 1/200 pregnancies in Caucasian women and can result in intrauterine fetal death, four heterozygous variants of FXR were found in or near the transcribed sequence of FXR being associated with downregulation of FXR target genes encoding for SHP and organic anion transporting polypeptide 1B3, upon BA stimulation [13,14]. Two different forms of inherited cholestasis, benign recurrent intrahepatic cholestasis (BRIC) and progressive familial intrahepatic cholestasis (PFIC1) were correlated with mutations in ATP8B1 (FIC1), in chromosome 18 [15], a membrane protein that activates FXR via protein kinase C-zeta, and mutations in FIC1 are associated with downstream effects of FXR on BA homeostasis [16]. Specifically, gain of function experiments in cells in vitro demonstrated that FIC1 overexpression enhanced FXR phosphorylation and nuclear localization as well as upregulation of *Bsep* which is transactivated by FXR. FIC1 effect was dependent on protein kinase C-zeta and also on CDCA presence in culture medium of the cells [16]. BRIC was related to a partially functional FIC1, and it has been suggested that it is caused by a deficient FIC1/PKC-zeta/FXR signaling pathway [16]. Progressive familial intrahepatic cholestasis (PFIC) is a liver injury characterized by early onset of BA accumulation in the liver, accompanied by symptoms of pruritus and malabsorption. In PFIC patients, genetic analysis identified inborn errors in CYP7A1 gene [17], suggesting formation of toxic bile due to predominant alternative biosynthesis of BA.

Table 1 summarizes the newest targets for drugs against cholestasis based on mechanistic studies on FXR and its role in BA homeostasis.

### 2.1. Targeting FXR for BA Regulation in Cholestasis

The role of FXR in the regulation of hepatic triglyceride and glucose homeostasis [6,55,56] has been well described and several FXR agonist drugs have been developed for treating dyslipidemia, nonalcoholic fatty liver disease (NAFLD), nonalcoholic steatohepatitis (NASH), and to improve liver functions [6]. However, the beneficial effects of FXR agonists in the treatment of liver fibrosis caused by biliary cholestasis, have been controversial. In cholestasis, the activation of FXR by excessive amounts of BA accumulated in the liver, is at a maximum, and it was demonstrated that administration of CDCA or DCA in their natural form, does not improve liver fibrosis in animal models of hepatic cholestasis [6]. Semi-synthetic BA and non-steroidal agonists of FXR have been developed and tested for therapies of liver and pancreas-related diseases [6]. Thus, INT747 or obeticholic acid (OCA), a 6-α-ethyl derivative of CDCA were proposed to have hepatoprotective effects on certain types of cholestasis based on animal model experiments [21,25]. Non-steroidal agonists of FXR such as GW4064 and WAY-362450, which may modulate multiple G protein-coupled receptors besides directly activating FXR, have been proposed to be used to reduce hepatic inflammation in the context of cholestasis [22,26,57].

Searches for FXR ligands on the database clinicaltrials.gov show numerous studies aiming to apply FXR ligands for many types of diseases including cholestasis but also alcohol-, obesity-, metabolic syndrome-, and diabetes-related liver injuries (e.g., alcoholic hepatitis, NASH, and NAFLD). Out of the total number of clinical trials listed, 12 studies used FXR agonist alone or in combination with either a fibrate such as benzafibrate or with ursodeoxycholic acid (UDCA), to treat biliary and cholestatic related conditions, and PBC in particular (Table 2). Recently, OCA was approved for use in combination with UDCA for the treatment of PBC [20], despite data suggesting that OCA has negative side effects exacerbating biliary injury in animal models of obstructive cholestasis [28]. In fact, in May 2021, Food and Drug Administration (FDA) published a safety communication that can be found on fda.gov, stating that FDA restricts use of OCA in PBC patients with advanced cirrhosis, due to risk of serious liver injury.

A recent review by Jiang et al. describes a full spectrum of small molecules including agonists, partial agonists, and antagonists of FXR, designed to be applied for BA-related liver diseases [58]. The article reveals not only a multitude of various 3D structures of the ligand binding domain of FXR upon binding different types of ligands, but also the structures of steroidal agonists (e.g., OCA, EDP-305, BAR502), nonsteroidal agonists (e.g., GW4064, Nidufexor, Cilofexor, Tropifexor/LJN452), partial agonist (TERN-101, DM175), as well as natural (ivermectin, tuberatolites,) and synthetic (T3, DY268, FLG249) antagonists of FXR [59]. While some of these ligands have shown positive results in clinical studies for treating cholestatic liver diseases, there are still challenges with negative side effects such as high incidence of increased serum LDL, reduced HDL, and pruritus [55,59]. New strategies have been developed to produce more efficient drugs with less adverse symptoms, for example a dual FXR and TGR5 agonist which regulates pathways from both receptors [58,60].

Besides its ability to control the transactivation of genes involved in BA biosynthesis and transport along the enterohepatic tract, and to contribute to liver regeneration and growth, FXR has been proved to counterbalance nuclear factor kappa-light-chain-enhancer of activated B cells (NF-kB)—mediated transcription of proinflammatory cytokines, having a role in hepatic repair in liver fibrosis [61,62,63]. Even though FXR is expressed also in HSC and cholangiocytes, it is critical in hepatocytes since BA-induced hepatocyte death activates profibrogenic factors such as transforming growth factor beta 1 (TGF-β1), platelet derived growth factor (PDGF), connective tissue growth factor (CTGF) which then act on quiescent HSC inducing their activation to proliferate and produce excessive extracellular matrix proteins and fibrosis [64,65]. Several published studies revealed that semi-synthesis and non-steroid agonist of FXR, e.g., GW4064 and WAY-362450, were able to mitigate liver inflammation and fibrosis in animal models of cholestasis [22,66,67].

FXR is expressed not only in the liver and entire gastrointestinal tract, but also at lower levels in kidneys, adrenals and even the brain [68,69,70,71]. It is important to underscore that depending on the specific expression of FXR within the body, its activation versus inhibition may favor or impair certain aspects of cholestasis and liver disease-associated syndromes. A good example is the evidence for FXR being expressed in cortical neurons and having role in molecular signaling of BA which induce neurological complications of liver disease [71,72]. It has been demonstrated in animal models of hepatic encephalopathy, that strategies to inhibit brain FXR signaling prevented accumulation of cholesterol in the cortex and neurologic decline [71]. Several dysfunctions in BA metabolism have been associated with neurodegenerative and neurological disorders, and therapies using BA with low hydrophobicity such as conjugated BA tauroursodeoxycholic acid (TUDCA) and UDCA which are considered FXR antagonists, have been found to be neuroprotective [73]. Therefore, future drugs aimed to modulate FXR activity for beneficial effects on the liver in hepatic cholestasis and fibrosis, will need to be designed to avoid unwanted side effects on other tissues and organs such as the brain.

### 2.2. Targeting FXR-Fibroblast Growth Factor (FGF) 15/19 Enterohepatic Pathway

The physiological role of FXR as master regulator of BA metabolism and homeostasis, was clearly demonstrated in FXR^−/−^ mice, which had abnormally large BA pool size and moreover, exhibited severe hepatotoxicity when challenged with cholic acid-rich diet [5]. It was determined that FXR is highly expressed in certain segments of the intestine, i.e., ileum and colon where it regulates the transcription of BA transporters and also hormones with role in BA homeostasis such as FGF15 [37,38]. As shown in Figure 1, BA-activated FXR in ileum upregulates FGF15/19 which is secreted into the portal circulation and acts on the liver where it inhibits BA synthesis by repressing Cyp7a1 gene in hepatocytes. FGF15/19 acts through a complex of FGF receptor 4 (FGFR4) and βKlotho protein which is a single transmembrane receptor with tyrosine kinase activity [74,75].

FXR gene deletion in mice demonstrated that FXR nuclear receptor plays a role in regulating hepatic inflammation, since FXR^−/−^ mice had high levels of proinflammatory cytokines such as interleukin 1 beta (IL-1β) and were prone to developing liver cancer due to abnormal expression of β-catenin and c-Myc oncogenes [76,77,78]. As mentioned by other reviews on the large array of FXR functions, it is interesting that selective intestinal FXR reactivation in FXR^−/−^ mice, protected against hepatocellular carcinoma [63]. Thus, intestinal FXR restores the control of FGF15 pathway-mediated BA synthesis, and ensures limitation of BA overload and prevention of hepatic inflammation and carcinogenesis [79].

It is presumed that the human ortholog of mouse FGF15 is FGF19, based on 53% amino acid identity; however, there is no definitive consensus on the matter [38]. It has been shown that in humans, serum FGF19 reaches a peak 90–120 min after postprandial release of BA, but before the repression of BA synthesis. Clinical studies indicated that treatment of healthy volunteers with BA sequestrants such as cholestyramine, reduced circulating FGF19, and conversely, administration of FGF19 analogues repressed BA synthesis. Thus, it was confirmed the negative feedback loop of FXR/FGF19 within ileum and the liver, to maintain BA homeostasis. Interestingly, patients suffering from BA-caused diarrhea who overproduce BA, have low circulating FGF19, suggesting a dysfunction of FGF19 and inability to inhibit CYP7A1 and BA synthesis.

The use of FGF19 analogues for the treatment of liver cholestasis appears as an attractive venue to develop new therapeutics, however FGF19 has been shown to promote cell growth and carcinogenesis [80]. A new therapeutic agent is NGM282, an FGF19 nontumorigenic variant that had been described to be hepatoprotective in nonalcoholic steatohepatitis [35], and is in clinical trial for PSC [36] and PBC therapy (NCT02135536) [80].

### 2.3. Targeting Hydrophobic BA with Role in Hepatocellular Death, Activation of HSC and Fibrogenesis

Cholestatic liver diseases are characterized by chronic hepatic and systemic accumulation of total BA and especially hydrophobic bile salts due to a dramatic dysregulation of BA homeostasis [81]. Most of the cholestasis syndromes are correlated with dysfunctions of genes encoding for transporters of BA along the hepato-biliary tract, such as ATP8B1 coding for phospholipid flippase [82], and ABCB11 for bile salt export pump or BSEP [83,84]. Early studies on the role of excessive BA in liver injury, pointed out that hydrophobic BA such CDCA and GCDC are toxic and induce hepatocellular death by apoptosis [85,86].

Liver injury caused by cholestasis involves a multitude of signaling pathways starting with hepatocyte damage or death, activation of Kupffer cells, recruitment of circulating monocytes and macrophages, activation of cholangiocyte and HSC. Therefore, for long time it has been difficult to assess the direct effect of hydrophobic BA on liver fibrosis, in vivo. However, recently, Hohenester et al. have used Atp8b1^G308V/G308V^ mice which have normal hepatic function except when are being fed high amounts of toxic BA and develop cholestasis [87]. This particular mouse model has been created after the G308V/G308V mutation of Atp8b1 gene was identified in humans susceptible to cholestasis [88]. Upon feeding on GCDC-rich diet, the Atp8b1^G308V/G308V^ mice exhibited pro-fibrotic markers including deposition of excess collagen, proliferation and activation of HSC. Interestingly, treatment of HSC isolated from Atp8b1^G308V/G308V^ mice, with CDCA hydrophobic BA, increased BrdU incorporation in proliferating cells and the expression of alpha smooth muscle actin (αSMA), while a more hydrophilic BA, UDCA did not induce profibrogenic effects [87]. It has been suggested that CDCA was able to induce epidermal growth factor receptor (EGFR)/mitogen-activated protein kinases (MEK)-dependent signaling pathway resulting in activation of extracellular signal-regulated kinase (ERK) followed by collagen deposition and cell proliferation [87]. It has been demonstrated that inhibitors of EGFR/MEK/ERK (i.e., AG1478, UO126 and PD98059, respectively) impaired HSC proliferation and collagen secretion induced by CDCA [87]. Thus, this particular signaling pathway in HSC may be target for future drugs, designed to inhibit EGF-induced proliferation of HSC and to lower liver fibrosis in cholestatic patients.

A strong emphasis is placed on studying potential drugs targeting BA biosynthesis, metabolism, and excretion. Rifampicin has been used firstly as an antimicrobial drug, and later on for the treatment of pruritus caused by biliary cholestasis, based on clinical observations. Later on, it was established that rifampicin (also known as Rifadin, Rimactane commercial names), is a drug that upregulates the expression of detoxification enzymes involved in BA metabolism [41,47,89]. Mechanistic studies indicated that rifampicin induces a number of drug-metabolizing enzymes (DME), with a prominent effect on the expression of cytochrome P450 (CYP) 3A4 in the liver [44]. In a study in which rifampicin was administered to BDL-rats and to human hepatocytes pre-treated with GCDCA, it was determined that phase I and II DME (including CYP3A4, CYP7A1, UDP-glucuronosyltransferase 2B4) and BA transporters (such as MRP4, BSEP) were increased while the import of BA into hepatocytes via Na^+^ taurocholate co-transporting polypeptide, NTCP, was reduced [90]. Clinical studies indicated that rifampicin was effective in treating cholestasis, and analysis of BA excretion suggested that rifampicin stimulated elimination of 6α-,1β, 2β, and 4β-hydroxyl BA in cholestatic patients during the treatment [91]. A study that looked at the effect of rifampicin on anion exchanger 2 (AE2) which mediates Cl^−^/HCO^3−^ exchange, concluded that the increase in bile flow induced by rifampicin is mainly due to increased HCO^3−^ excretion mediated by increased expression of AE2 [51]. Various mechanisms underlying the choleretic effects of rifampicin are still to be investigated.

Based on publications in reference to metabolites in the mevalonate pathway being related to the hepatic metabolism of BA, a new study demonstrates that conditional deletion of the enzyme that synthetizes geranylgeranyl pyrophosphate (GGPP) from farnesyl pyrophosphate (FPP) in the liver, decreases the level of hepatic BA [48]. This discovery may lead to designing efficient inhibitors of geranylgeranyl diphosphate synthase (GGPPS) to be applied as medication for the alleviation of obstructive cholestatic disease [48].

More recent investigations focused on a possible role of the short form of augmenter of liver regeneration (sfALR) in preventing BA synthesis and BA-induced apoptosis in hepatocytes [40,92]. ALR, also known as hepatopoietin, was discovered as a growth factor with role in hepatocyte proliferation after liver injury, and then shown to be expressed ubiquitously in all organs and exclusively in hepatocytes, in the liver [39]. An initial study on the effect of toxic BA on ALR expression, revealed that even though Alr gene is upregulated at transcriptional level by early growth response-1 protein (Egr-1), BA suppress ALR transcription independently of Egr-1, but via hepatocyte nuclear factor 4-alpha (HNF4α)/SHP pathway [92]. It has been shown that human hepatoma cells with high expression of sfALR in the cytoplasm, exhibited reduced Cyp7a1 mRNA level and lower production of BA, and this was attributed to activation of signal transducer and activator of transcription 3 (STAT3) [39]. Overexpression of sfALR in hepatoma cells caused significant reduction in GCDA-induced apoptosis. Consistent with sfARL being an inhibitor of BA effects on hepatocytes, it was also found that human cholestatic liver samples had low mRNAs of ALR and forkhead box protein A2 (FOXA2) also known as hepatocyte nuclear factor 3α (HNF-3β), a positive transactivator of ALR gene [39]. FOXA2 has important roles to enable access to closed chromatin and displace linker histones, promoting cells type specification [93,94]. These data suggest that ALR, FOXA2 and STAT3 may be drug targets for decreasing BA biosynthesis and subsequent fibrogenesis in cholestatic patients.

## 3. Targeting FXR in Portal Hypertension Associated with Cholestasis-Induced Cirrhosis

Cirrhosis is the final stage of chronic cholestasis, in which hepatic fibrosis is very advanced so that most of the liver functions are impaired [95]. Thus, advanced hepatic inflammation, biliary and periportal fibrosis, loss of tissue homeostasis followed by abnormal remodeling are conducive to late permanent dysfunctional state of cirrhosis [96]. Portal hypertension (PHT) is commonly seen in patients with cholestatic liver cirrhosis especially in the stage of decompensation when liver injury is associated with complications such as ascites, hepatic encephalopathy, or variceal bleeding [97]. FXR has been found to be a promising target for the treatment of portal hypertension. Thus, in liver fibrosis, the blood vessels are more constricted compared to normal liver due to a significant decrease of FXR activation caused by ROS and proinflammatory cytokines, resulting in suppressed endothelial nitric oxide synthase (eNOS) and nitric oxide (NO) [98]. Vascular FXR in general was demonstrated to be an important regulatory factor, since pharmacological and genetic activation of FXR stimulated eNOS promotor activity [98]. For the treatment of portal hypertension in particular, Mookerjee et al. studied the effect of an FXR agonist, OCA, on dimethylarginine-dimethylaminohydrolase1 (DDAH-1), a marker of portal pressure that is expressed in hepatocytes and downregulated in cirrhosis [99]. It was shown that asymmetric-dimethylarginine (ADMA), an eNOS inhibitor which is metabolized by DDAH-1 was significantly reduced upon treatment with OCA in an animal model of cholestatic cirrhosis, due to rescue of DDAH-1 expression via activation of FXR [99].

Another interesting study was performed on PX20606 (PX), a nonsteroidal agonist of FXR, in regard to its effect on portal hypertension besides liver fibrosis in experimental models of non-cirrhotic (partial portal vein ligation or PPVL) and cirrhotic (carbon tetrachloride, CCl4) models [100]. PX was able to decrease portal pressure markers in both non-cirrhotic and cirrhotic rats, confirming that FXR has critical role in the regulation of eNOS and portal pressure in the liver.

## 4. FXR Involvement in Autophagy during Cholestasis

Recent studies on hepatic autophagy revealed an important function of this process in maintaining the overall homeostasis of the liver. Autophagy is an evolutionary conserved mechanism of lysosome-dependent degradation of intracellular components, with multiple functions including cell energy homeostasis, organelle turnover, clearance of aggregated materials inside cells and defense against intracellular pathogens [101]. Deficiencies in autophagy result in several pathologies associated with hepatomegaly, liver inflammation and fibrosis and even carcinogenesis [101]. It was first demonstrated that in BDL mice, cholestasis was associated with hepatocyte autophagy activation [102]. In these studies, suppression of autophagy with chloroquine increased hepatocyte apoptosis, while activation of autophagy with rapamycin decreased cholestatic liver injury, and it was concluded that autophagy benefited hepatocyte survival via modulation of reactive oxygen species (ROS) [102]. Later on, it was found that the accumulation of a protein p62/SQSTM1 (sequestosome 1) disabled the ubiquitination and degradation of nuclear factor-erythroid 2-related factor 2 (NRF2), leading to liver injury [103,104,105]. Generally, NRF2 is known as a transcription factor that targets genes with role in adaptive protection against oxidative stress in cells [106]. It was also suggested that NRF2 is involved in the regulation of autophagic processes in response to oxidative stress, functioning in a negative feedback loop in opposition to AMP-activated protein kinase (AMPK), which is critical for autophagy induction via mTOR downregulation (mTOR or mammalian target of rapamycin, has role in cell growth and autophagy) [107]. Recently, it was determined that increased NRF2 due to defective autophagy, causes a larger release of high mobility group box 1 (HMGB1), a protein released during necrosis) from hepatocytes and consequently, enhanced the ductular reaction [105]. Moreover, NRF2 is related to the dysfunction of BA synthesis, secretion and regulation, affecting the expression of FXR, the main nuclear receptor that regulates BA metabolism and transport within the liver [101]. Thus, Khambu et al. [101] demonstrated that mice deficient in autophagy due to a lack of Atg7 and Atg5 genes, exhibited hepatic cholestasis characterized by increased serum and liver BA loads, biliary hyperplasia, and suppressed BA transporters such as BSEP which are transactivated by FXR. Interestingly, deletion of Nrf2 gene in autophagy-deficient mice, rescued FXR suppression and reversed the cholestatic injuries [101]. The authors concluded that there is a regulatory loop between FXR and autophagy, in which BA can suppress autophagy, and deficiency in autophagy downregulates FXR via NRF2 expression [101]. This study suggests that several targets including AMPK, NRF2, autophagy regulators and FXR are to be considered for developing novel therapies for liver cholestatis and fibrosis. Thus, betulinic acid [53] (a natural pentacyclic triterpenoid), 5-aminoimidazole-4-carboxamide-1β-D-ribofuranoside (AICAR) [108], metformin [109,110], and GSK621 [111] are AMPK activators known for beneficial effects related to several diseases including diabetes, obesity, chronic inflammation, and cancer, and deserve to be investigated in the context of cholestasis [112]. In fact, recent studies identified the AMPK/FXR axis as having critical role in cholestatic liver injuries [113]. NRF2 transcription factor has been considered to be almost exclusively positive in promoting cell survival under detrimental conditions due to increased reactive oxygen radicals, via activation of target genes bearing antioxidant response element (ARE) in their promoters [31]. However, there are also negative effects related to NRF2, for example, excessive upregulation of NRF2 pathway can result in cell dysfunction or help cancer cell survival and chemotherapy resistance [29]. A quest for NRF2 antagonists led to the identification of brusatol, a quassinoid isolated from an evergreen shrub *Brucea javanica*, to decrease the level of NRF2 in a series of cancer cell lines [29,30]. The effect of such NRF2 inhibitors on models of cholestatic liver injuries, are still to be tested.

**Figure 2:**
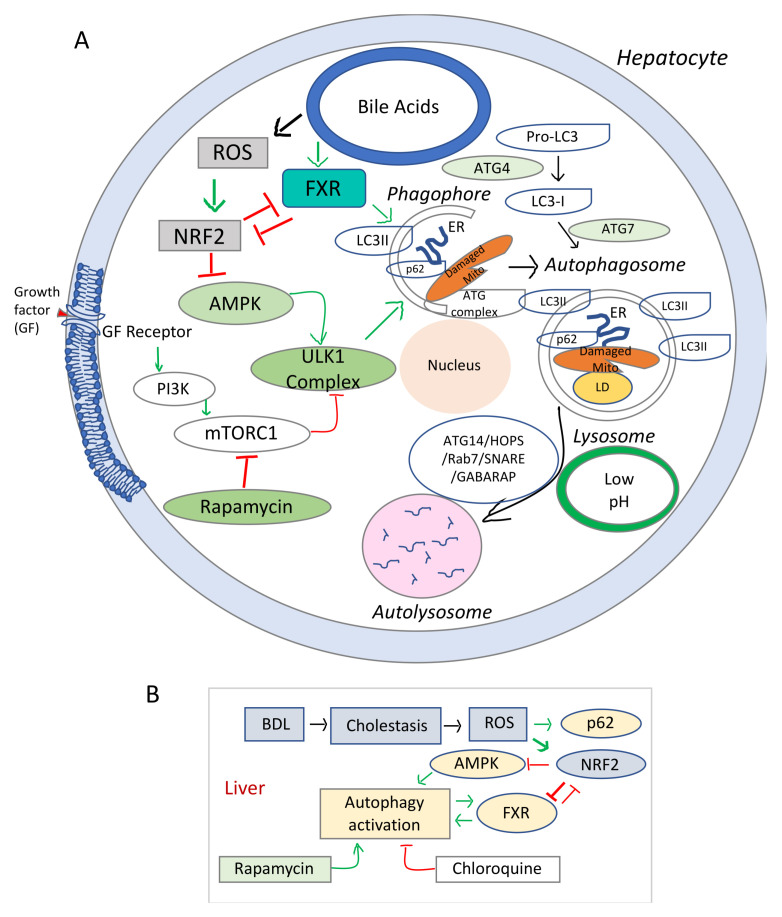
Diagram of signaling pathways involved in the dysregulation of autophagy and cell growth in cholestasis and liver fibrosis. (**A**), Schematic representation of the elements involved in the autophagy dysfunction in chronic oxidative stress. mTOR (mechanistic target of rapamycin) is a serine/threonine protein kinase, component of two distinct cellular complexes termed mTOR complex 1 (mTORC1) and mTORC2 where a multitude of signals such as nutrients, growth, energy are sensed and processed [114]. mTORC 1 downregulates autophagy (a catabolic process where macromolecules are sequestered in double membrane bound autophagosomes that fuse with lysosomes to allow their enzymatic breakdown). When there is sufficient energy, mTORC1 is active and through phosphorylation and destabilization of autophagy promoting complexes (ULK1 and other proteins), it ensures that autophagy is inhibited. During conditions of starvation, mTORC1 is inactive, it allows the phagosome to be formed. In addition, due to low energy level, AMPK gets activated and phosphorylates ULK1. Sequestome 1 (SQSTM1) or p62 is an active component of the autophagic process, and acts as a scaffold on lysosomal membranes for mTOCR1. During autophagy, p62 is incorporated into autophagosome and degraded, thus limiting mTORC1 activity. LC3 or microtubule-associated proteins 1A/1B light chain 3B is a biomarker of autophagosome. (**B**), Diagram of the interaction of cholestasis-induced ROS with autophagy in hepatocytes. While stimulating p62 and phagosome formation, ROS inhibit AMPK-mediated energy production needed for the autophagic flux, via NRF2 upregulation. Stimulation of autophagy by rapamycin and overexpression of FXR in autophagy-deficient cells counteract the effects of NRF2 reducing ROS-mediated hepatic damage.

The schematic representation in Figure 2 summarizes the main signaling pathways involved in the regulatory process of autophagy in relation to hepatocyte injuries due oxidative stress. The signaling pathways initiated by BA through FXR, HNF4α, and other transcription factors and the effects of BA on various homeostatic processes such as autophagy are still to be understood (i.e., how increased BA in the liver due to biliary obstruction, influence cell energy homeostasis, autophagy and lysosome functions). Data from studies on the effect of FXR agonist on liver cholestasis, have been controversial, probably due to a poor understanding of the FXR role in autophagy.

In summary, recent studies show that increased ROS as result of BA toxicity in cholestasis, have negative effects on autophagy, and more drugs are to be designed to address the signaling pathways of FXR in connection to autophagy.

## 5. Conclusions

Currently, there are only few therapies for the treatment of cholestasis and liver fibrosis, including UDCA, nor-UDCA, OCA, and rifampicin. The first-line therapy in most cholestasis disorders is treatment with UDCA, the hydrophilic BA which enhances hepatobiliary flow, being effective in approximately 50% of patients of most forms of cholestasis. Recently OCA was approved as a second-line of therapy for patients who do not respond to UDCA treatment. Another FXR agonist, i.e., GW4064 which demonstrated decreased BA synthesis in preclinical studies, was not approved for clinical trials due to the short terminal half-life of the drug. Rifampicin (Rifadin, Rimactane commercial names) is a drug that upregulates the expression of detoxification enzymes involved in BA metabolism.

## Figures and Tables

**Table 1:** New drug targets related to BA homeostasis and FXR for treating cholestasis-induced hepatic fibrosis.

Dysfunction	Signaling Pathway/Genetic Defect	Drugs	References
Reduced FXR activity in hepatocytes	FXR/SHP/BA synthesis enzymes	Natural (UDCA) and synthetic (INT747, GW4046, WAY-62450) FXR ligands	[6,20,21,22,23,24,25,26,27,28]
Reduced FXR expression in hepatocytes	ROS/NRF2/FXR	-Inhibitors of NRF2: Brusatol;	[29,30,31]
		-Inhibitors of ROS: N-acetyl cysteine	[32,33,34]
Reduced expression of FXR in hepatocytes	FGF19/Src/FXR	FGF19-induced activation of Src to phosphorylate FXR	[35,36,37,38]
Excessive hydrophobic BA in hepatocytes	HNF4α/SHP/ALR (hepatopoietin)	Activation of ALR, FOXA2, STAT3	[39,40]
Excessive total BA in hepatocytes	-Reduced expression/activity of enzymes that catabolize BA;	-Inducers of drug-metabolizing enzymes: CYP3A4, CYP7A1;	[41,42,43,44,45,46,47]
	-Increased expression/activities of enzymes of BA biosynthesis;	-Inhibitors of GGPP, FPP;	[48]
Impaired BA homeostasis	BA transport proteins:	-Inducers of BA exporters (BSEP, MRP4) and HCO_3_- excretion (AE2);	[49,50,51]
		-Inhibitors of NTCP (recovers BA from intestine): rifampicin	[47,51]
Impaired autophagy	AMPK/Autophagy/FXR	-Activators of AMPK: betulinic acid, AICAR	[52,53]
Impaired autophagy	ROS/NRF2/Autophagy/FXR	-Inhibitors of ROS-generating oxidative enzymes	[33,54]

**Table 2:** Clinical trials for testing FXR ligands or FXR-related targets in PBC/PSC patients, as listed in clinicaltrials.gov database.

Study	NCT Number	Conditions	Treatments
OCA in PSC cholangitis	NCT02177136	PSC	OCA vs. Placebo
A post-authorization noninterventional observational of patients with PBC cholangitis treated with OCA in real time.	NCT03703076	PBC	OCA vs. Placebo
Phase 4 study of OCA evaluating clinical outcomes in patients with PBC	NCT0238111	Liver cirrhosis, biliary	OCA vs. Placebo
Prospective, multicenter cohort study on PBC	NCT04076527	PBC	UDCA vs. Ocaliva
Study of OCA evaluating pharmacokinesis and safety in patients with PBC and hepatic impairment	NCT03633227	Liver cirrhosis, biliary	OCA vs. Placebo
Study of OCA in combination with BZF evaluating efficacy, safety, and tolerability in patients with PBC	NCT04594694	Liver, cirrhosis, biliary	OCA + BZF vs. OCA only, BZF only, Placebo
Linerixibat and OCA drug interaction study in healthy subjects	NCT04053023	Cholestasis	GSK2330672 + OCA vs. each drug alone, or Placebo
Phase 3 study of OCA in patients with PBC	NCT01473524	PBC	OCA vs. Placebo
OCA in bariatric and gallstone disease	NCT01625026	Gall stones, obesity	OCA vs. Placebo
Effect of OCA on transport of BA in PBC examined by 11C-cholyl-sarcosine PET/CT	NCT03253276	PBC	OCA vs. Placebo
Study of INT 747 in combination with URSO in patients with PBC	NCT00550862	PBC	INT-747, URSO/UDCA vs. Placebo
Study of OCA combination with UDCA in patients with PBC	NCT04956328	PBC	OCA + UDCA vs. Placebo, OCA only, UDCA only.
Study of INT-747 as monotherapy in participants with PBC	NCT00570765	PBC	INT-747 vs. Placebo
